# Repellency, prevention of attachment and acaricidal efficacy of a new combination of fipronil and permethrin against the main vector of canine babesiosis in Europe, *Dermacentor reticulatus* ticks

**DOI:** 10.1186/s13071-015-0682-z

**Published:** 2015-01-27

**Authors:** Pascal Dumont, Josephus J Fourie, Mark Soll, Frédéric Beugnet

**Affiliations:** Merial S.A.S., 29 Av Tony Garnier, 69007 Lyon, France; ClinVet International (Pty) Ltd, PO Box 11186, 9321 Universitas, South Africa

**Keywords:** Repellency, Acaricidal efficacy, *Dermacentor reticulatus*, Ticks, Fipronil, Permethrin

## Abstract

**Background:**

*Dermacentor reticulatus* is a European hard tick of major veterinary importance because it is the vector of canine babesiosis due to *Babesia canis*. The efficacy against this particular tick species is therefore a key characteristic for an acaricidal solution for dogs. The repellency, prevention of attachment and acaricidal efficacy of Frontline Tri- Act®/Frontect®, a new combination of fipronil and permethrin against induced infestations of *Dermacentor reticulatus* ticks on dogs were evaluated after a single topical administration.

**Methods:**

A group of 20 dogs were allocated to two treatment groups. Ten dogs were treated with a topical spot-on formulation containing 6.76% w/v fipronil + 50.48% w/v permethrin once on Day 0 and 10 dogs served as untreated controls. Tick infestations were performed by placing 50 *D. reticulatus* ticks next to sedated dogs confined to infestation crates on days 1, 7, 14, 21 and 28. Thumb counts on dogs were conducted at 4, 12 and 24 h post-challenge. Tick removal counts were performed 48 h after each infestation. Repellency, prevention of attachment and acaricidal efficacy were calculated.

**Results:**

The new combination provided repellency ranging between (56.5–73.5%) at 4 h post-infestation (pi), between (76.3–92.9%) at 12 h pi and between (83.9–96.5%) at 24 h pi, up to 4 weeks post-treatment. Prevention of attachment ranged between (64.1–79.7%) at 4 h pi, between (79.1–94.2%) at 12 h pi and between (84.2–99.6%) at 24 h pi, up to 4 weeks post-treatment. Acaricidal efficacy against *D. reticulatus* ticks was ≥99.5% for 4 weeks post-treatment.

**Conclusion:**

The new combination of fipronil and permethrin demonstrated excellent repellency, prevention of attachment and acaricidal efficacy against *D. reticulatus* for at least 4 weeks. The results suggest that in endemic areas of canine babesiosis, the application of the new combination can significantly reduce the potential for transmission of *B. canis* as well as other tick-borne diseases.

## Abrégé

**Contexte:**

*Dermacentor reticulatus* est une tique européenne d’importance vétérinaire majeure car c’est le vecteur de la babésiose canine due à *Babesia canis*. L’efficacité contre cette espèce de tique est donc une caractéristique clef pour un médicament acaricide pour chien. L’activité répulsive, la prévention d’attachement et l’efficacité acaricide de Frontline Tri- Act®/Frontect® (Merial), nouvelle combinaison de fipronil et perméthrine, ont été évaluées contre des infestations expérimentales de chiens par des tiques *Dermacentor reticulatus*, après une application topique unique.

**Méthodes:**

20 chiens ont été répartis en deux groupes. Dix chiens ont été traités une fois au jour 0 avec une formulation topique spot-on contenant fipronil à 6,76% w/v + perméthrine à 50,48% w/v et dix chiens ont servi de témoins non traités. Les infestations ont été réalisées en plaçant 50 tiques *D. reticulatus* à proximité des chiens tranquillisés et confinés dans des cages d’infestation, aux jours 1, 7, 14, 21 et 28. Des comptages par palpation ont été réalisés sur les chiens 4, 12 et 24 h post-challenge. Les comptages /retraits des tiques ont été réalisés 48 h après chaque infestation. L’activité répulsive, la prévention d’attachement et l’efficacité acaricide ont été calculées.

**Résultats:**

La nouvelle combinaison a conféré une activité répulsive de 56,5 à 73,5% 4 h post-infestation (pi), de 76,3 à 92,9% 12 h pi et de 83,9 à 96,5% 24 h pi, jusqu’à 4 semaines post-traitement. La prévention d’attachement a été de 64,1 à 79,7% 4 h pi, de 79,1 à 94,2% 12 h pi et de 84,2 à 99,6% 24 h pi, jusqu’à 4 semaines post-traitement. L’efficacité acaricide contre les tiques *D. reticulatus* a été ≥ 99,5% pendant 4 semaines post-traitement.

**Conclusion:**

La nouvelle combinaison de fipronil et perméthrine a démontré d’excellentes efficacités répulsive et acaricide ainsi qu’une excellente prévention d’attachement vis-à-vis de *D. reticulatus* pendant au moins 4 semaines. Ces résultats suggèrent que, dans les zones enzootiques pour la babésiose canine, l’application de la nouvelle combinaison pourrait significativement réduire le risque de transmission de *B. canis* tout comme celui d’autres maladies vectorielles à tiques.

## Background

In temperate areas of Europe, *Dermacentor reticulatus* is a quite common tick species affecting dogs and is the primary vector of canine babesiosis due to *Babesia canis* [1,2]. *D. reticulatus*, is a three-stage polytropic, and hydrophilic tick. Larvae and nymphs infest micro-mammals and are endophilic, living in their hosts burrows, whereas adults are exophilic with a tropism primarily for dogs and at a lower extent to ungulates such as horses, sheep and cattle. This tick is adapted to temperate climate with a strong preference for open areas with a high humidity level, especially riverbanks, paths sides, parkland and wasteland [3]. A strong correlation between *D. reticulatus* and canine babesiosis distribution is widely supported [4]. Canine babesiosis is a tick-borne disease caused by intraerythrocytic protozoa of the genus *Babesia* which can cause severe clinical illness [4-6]. In hyper-endemic area of Southern France the incidence of the disease reaches up to 16% of the dog population [4]. In recent years, the geographic distribution of babesiosis has expanded from western and central Europe toward northern Europe, probably due both to changes in the climate which has increased tick survival and due to an increase in companion animal travels [4-6].

Current drugs available to treat canine babesiosis (e.g., imidocarb dipropionate) do not completely eliminate the pathogen at the recommended dose and treated dogs may suffer a relapse or serve as a reservoir for the further spread of *Babesia* parasites in a given area [7]. Vaccination against *B. canis* is possible in some countries. It consists in administering soluble antigens grown organically from *B. canis*, added to an immunity adjuvant, saponin. The efficacy of the vaccine is not complete, it protects against the severity of the clinical signs [8]. In the absence of sufficient direct preventive measure, the use of acaricidal product with a sufficient and persistent speed of kill is recommended. In a given area, it is important to check the efficacy of the product against the specific tick species that transmit the disease.

Fipronil, a phenylpyrazole with both insecticidal and acaricidal properties, has been used for several years for the treatment and control of ticks, fleas and lice [9,10]. Permethrin, a synthetic pyrethroid, has both killing and repellent effect and is active against a wide range of pests including ticks, fleas, lice and various hematophagous Diptera [9,11].

The study reported here was conducted to assess the repellent and acaricidal efficacy of a new combination of fipronil and permethrin (Frontect®/Frontline Tri-Act®) against the main vector of canine babesiosis in Europe, *D. reticulatus* ticks.

## Methods

A laboratory study was conducted according to Good Clinical Practices (GCP) as described in International Cooperation on Harmonisation of Technical Requirements for Registration of Veterinary Medicinal Products (VICH) guideline 9. It was designed as a parallel group, randomized, blinded, controlled efficacy study, and in accordance with standard methods for evaluating the efficacy of parasiticides for the treatment, prevention and control of tick infestations [12].

### Animals

Animals used in this study were adult mongrel dogs which had not been exposed to ectoparasiticides within 12 weeks prior to treatment (10 males and 10 females, 1 to 8 years of age, weighing 7.4–21.8 kg). The study animals were kept individually in cages of 1.9 m × 2.97 m, and no contact between dogs was possible. The dog cages were part of an indoor animal unit, environmentally controlled for temperature (20 ± 4°C). Dogs were managed similarly and with due regard for their well-being. Animals were handled in compliance with Merial Ethical Committee and in compliance with the South African National Standard “*SANS 10386:2008 The care and use of animals for scientific purposes*”. The dogs were acclimated to study conditions for 7 days prior to treatment and were observed for general health conditions throughout the study.

### Allocation and treatment

Dogs were ranked by pre-treatment live attached tick counts within gender to ensure equal representation of males and females within each group. They were then randomly allocated to one of the two groups by blocks of two dogs. Animals in Group 1 (n = 10) were not treated and served as control dogs. Animals in Group 2 (n = 10) were treated on Day 0 with the new combination at the minimum recommended dose (0.1 ml/kg based on Day 0 body weight, corresponding to a dose of approximately 6.76 mg/kg fipronil and 50.48 mg/kg permethrin). The dose was applied by parting the hair and applying the formulation directly onto the skin on the midline of the neck. The total volume was divided into two approximately equal volumes. One fraction was applied between the base of the skull and the shoulder blades and the other fraction was applied at the front of the shoulder blades. Dogs were observed prior to treatment and hourly for 4 h following treatment administration.

### Tick infestations and counts

*D. reticulatus* ticks were from a laboratory bred strain of European origin. Ticks used in the artificial infestations were unfed adults, at least one week old, and had a balanced gender ratio (50% female: 50% male).

Tick infestations were performed with 50 (±5) ticks one day after treatment (Day 1) and then on Days 7, 14, 21 and 28. The dogs were sedated and placed into individual exposure crates and the ticks were placed onto the floor of the crate next to each dog’s back. The dogs were removed from the exposure crate after 2 h and placed into a second crate. The dogs were removed from the second crate after an additional 2 h and returned to their normal housing. Ticks were collected and counted *in situ* in the two crates after each dog was removed (i.e., 2 and 4 h post-infestation).

Thumb counts were also conducted on all dogs at 4, 12 and 24 h post-infestation (ticks were categorized as live free, live attached, dead free or dead attached). Ticks were removed from the dogs and counted 48 h after infestation (Days 3, 9, 16, 23 and 30) and categorized as dead/live and free/attached in accordance to the WAAVP guideline [12].

Examiners wore personal protective equipment and were blinded as to treatment groups.

### Data analysis

#### Counts of ticks in crates

The total number of ticks (dead and alive) collected *in situ* in the two crates for each dog was summed by control or treatment group and the proportion out of the total infested in each group (n = 500) was calculated. The total number of ticks in the crates from the treated group was compared to the number of ticks in the crates from the untreated control group using the chi-squared test. The testing was two-sided and used a significance level of 5%. The analyses were performed using SAS® Version 9.1.3.

#### Repellency, prevention of attachment and acaricidal efficacy on dogs

Repellency of ticks was based on the total count of ticks (live or dead, attached or free) observed on dogs from the thumb counts at 4, 12 and 24 h.

Prevention of attachment was based on the total count of attached ticks (live or dead) from the same thumb counts at 4, 12 and 24 h.

Acaricidal efficacy on dogs was calculated from the total counts of live ticks removed from dogs 48 h after the start of each infestation.

For each variable (repellency, prevention of attachment and acaricidal efficacy), the total count per dog was transformed to the natural logarithm of (count + 1) for calculation of geometric means by group at each time point. Percent reduction from control was calculated for the treated group at each post-treatment time point using the formula [(C – T)/C] × 100, where C is the geometric mean for the control group and T is the geometric mean for the treated group. The treated group was compared to the untreated control group using the Friedman rank test with blocks defined as the allocation blocks. The testing was two-sided and used a significance level of 5%. The analyses were performed using SAS® Version 9.1.3.

## Results

The total number of ticks that were collected *in situ* in crates ranged from 7 to 22 in the control group and from 178 to 294 in the treated group, representing from 1 to 4%, and from 36 to 59% out the 500 ticks used for infestation in each group, respectively (Table 1). At all time-points and on all assessment days, significantly fewer ticks were recorded in crates from the untreated control group compared to the treated group (*p* < 0.002).

**Table 1:** **Number of** ***D. reticulatus*** **collected** ***in situ*** **into the crates for the two treatment groups at 2 and 4 h post-infestation (proportion out of the 500 ticks used for infestation)**

	**Untreated control group number of ticks in the crate** ***(percent out of the total number of ticks)***	**Treated group number of ticks in the crate** ***(percent out of the total number of ticks)***
Number of ticks and dogs Infestation Day	500 ticks/10 dogs	500 ticks/10 dogs
1	12 *(2%)*	178 *(36%)*
7	7 *(1%)*	294 *(59%)*
14	9 *(2%)*	271 *(54%)*
21	22 *(4%)*	262 *(52%)*
28	8 *(2%)*	203 *(41%)*

*Number of ticks differed statistically significantly between treatment groups at each time count (*p *< 0.002).*

The geometric mean tick counts for the untreated control group ranged from 30.8 to 35.9 during the 24-hour thumb counts (Table 2), and from 31.2 to 35.9 at the 48-hour removal and counts (Table 3). At all time-points and on all assessment days, significantly fewer ticks were recorded on the treated group compared to the untreated control group (*p* < 0.002).

**Table 2:** **Percent repellency and prevention of attachment of** ***D. reticulatus*** **in dogs treated with the new combination of fipronil and permethrin from thumb counts at 4, 12 and 24 h post-infestation**

		**Mean number of ticks (live or dead, attached or free) on dog**		**Repellency (%)**	**Mean number of attached ticks counted on dog**		**Prevention of attachment (%)**
**Infestation day**	**Time post-infestation**	**Untreated control group (10 dogs)**	**Treated group (10 dogs)**		**Untreated control group (10 dogs)**	**Treated group (10 dogs)**	
1	4 h	39.1	14.6	**62.5**	37.4	13.0	**65.2**
7		34.7	9.2	**73.5**	32.9	7.4	**77.6**
14		34.5	9.5	**72.5**	31.9	6.5	**79.7**
21		35.3	12.7	**63.9**	32.6	8.8	**72.9**
28		35.6	15.5	**56.5**	33.3	12.0	**64.1**
1	12 h	35.5	8.4	**76.3**	35.3	7.4	**79.1**
7		33.5	2.4	**92.9**	32.8	1.9	**94.2**
14		36.8	3.6	**90.3**	36.2	3.0	**91.7**
21		36.5	7.1	**80.5**	36.1	5.5	**84.7**
28		33.1	6.7	**79.8**	32.6	5.2	**84.1**
1	24 h	32.5	5.2	**83.9**	32.4	5.1	**84.2**
7		33.3	1.2	**96.5**	33.0	0.1	**99.6**
14		35.9	1.6	**95.5**	35.7	1.1	**96.8**
21		32.8	3.4	**89.7**	32.6	1.6	**95.0**
28		30.8	1.9	**93.7**	30.8	1.8	**94.1**

*Number of ticks differed statistically significantly between treatment groups (*p *< 0.002).*

**Table 3:** **Percent acaricidal efficacy of the new combination of fipronil and permethrin at 48 h post-infestation against** ***D. reticulatus*** **on dogs**

	**Mean number of live ticks counted on dog**		**Acaricidal efficacy (%)**
**Infestation day**	**Untreated control group (10 dogs)**	**Treated group (10 dogs)**	
1	32.5	0.1	**99.6**
7	35.9	0.0	**100.0**
14	34.7	0.0	**100.0**
21	33.7	0.0	**100.0**
28	31.2	0.1	**99.5**

*Number of ticks differed statistically significantly between treatment groups (*p *< 0.002).*

Percent repellency of the new combination was ≥56.5% (56.5–73.5%) at 4 h post-infestation, ≥76.3% (76.3–92.9%) at 12 h post-infestation and ≥83.9% (83.9–96.5%) at 24 h post-infestation for 4 weeks post-treatment (Table 2).

Prevention of attachment was ≥64.1% (64.1–79.7%) at 4 h post-infestation, ≥79.1% (79.1–94.2%) at 12 h post-infestation and ≥84.2% (84.2–99.6%) at 24 h post-infestation for 4 weeks post-treatment (Table 2).

Acaricidal efficacy against *D. reticulatus* was ≥99.5% for 4 weeks post-treatment (Table 3).

Dogs were in good health throughout the study with no health observations related to the treatment and no concurrent medications other than the sedative given during the study.

## Discussion

This study demonstrated the repellent effect, the prevention of attachment and the acaricidal efficacy of a new combination of fipronil and permethrin against *D. reticulatus* ticks on dogs.

The repellent effect is defined in the EU “Guideline for the testing and evaluation of the efficacy of antiparasitic substances for the treatment and prevention of tick and flea infestation in dogs and cats” (EMEA/CVMP/005/2000 – Rev.2) as: “*no tick will attach to the animal or no ticks should be detectable on the animal after 24 h following administration of the product*”. The 24-hour tick counts confirmed the excellent repellent effect of the new combination against adult, unfed *D. reticulatus* ticks for at least 4 weeks after treatment (≥93.7% repellency, except for the first infestation with 83.9% and at Day 22 with 89.7%, and ≥ 94.1% prevention of attachment, except for the first infestation with 84.2%). With another design, Dryden *et al.* [13] assessed the repellency of the combination permethrin + imidacloprid against *D. variabilis* and found the repellency to be 93.0% on Day 1 and then to range from 86.0 to 49.0% from Day 7 to Day 28.The lower repellent efficacy observed after the first infestation post treatment is probably to correlate to the diffusion of the actives on the skin, which may not be totally completed within 48 h.

Several publications have pointed out the difficulty to evaluate repellent efficacy against crawling arthropods and especially for those with a long-lasting host-parasite association, such as ticks [12,14] and the latest WAAVP guideline proposed a method for repellency evaluation that would be to place sedated treated and control dogs in infestation crates and to release ticks in the crates (not on the dogs) and only count the ticks remaining in crates [12]. A modification of this methodology was thus used in the present study by counting the ticks in the crate 2 h and 4 hours post infestation. The number of ticks collected in crates at 2 and 4 h post-infestation provides another way to assess repellency and the high numbers in the treated group (from 36 to 59% of ticks used for infestation) did support the repellency based on the tick count assessment on dogs. The very low numbers in the control group (from 1 to 4%) did support the robustness of the challenge with nearly all ticks being attracted by the untreated dogs.

Prevention of attachment, which is important to avoid tick feeding and, hence, pathogen transmission, was demonstrated as early as the 4-hour and 12-hour time points with ≥ 64.1% and ≥ 84.1%, respectively, from the Day 7 to Day 28 infestations, indicating that most of the ticks would not have time to transmit *Babesia*. It is estimated that *Babesia* spp. are mainly transmitted between 36 to 72 h after attachment [15].

The acaricidal efficacy of the new combination was remarkably high (≥99.5%) for the duration of the study compared to another topically applied formulation of permethrin combined with imidacloprid [16,17]. The new combination was effective immediately (99.6% efficacy on Day 1 after treatment). The permethrin + imidacloprid product, on the other hand, was reported to have a curative acaricidal efficacy of 81.2%, 82.6% and 93% in three different studies [16,17]. The acaricidal efficacy of the fipronil and permethrin combination remained ≥99.5% for 4 weeks, while the efficacy of the permethrin + imidacloprid product ranged from 86.5% to 93.0% on Day 7, from 76.8% to 97.0% on Day 14, from 73.4% to 87.0% on Day 21, and from 17.5% to 76.0% on Day 28 [15-17]. In addition, according to the labels of several permethrin-based marketed product, *D. reticulatus* appears to be less susceptible to permethrin than other tick species. This is illustrated by a shorter duration of efficacy against *D. reticulatus* than against *Ixodes ricinus* or *Rhipicephalus sanguineus*.

The two active ingredients of the new combination are well known for their acaricidal efficacy [9,16-20] and their combined acaricidal effect likely explains the high acaricidal activity of the product as well as the longer duration of acaricidal effect.

The study design used in the study was similar to the one used in Prullage *et al.* [21]. Ticks were placed next to the dogs and not in the fur; hence, the ticks had to move toward the host, which can be considered more similar to natural conditions. The thumb counts at 4, 12, and 24 h, although less accurate than a removal and count because they do not allow an easy counting by isolating the tick from the dog, gave data on the repellent effect while maintaining the ticks on dogs for the 48 h acaricidal efficacy assessment. The high number of ticks observed in the untreated control group at all time-points indicates that the tick challenge was vigorous on all assessment days (>30 ticks corresponding to >60% of the ticks used for infestation).

## Conclusions

The new combination of fipronil and permethrin provides excellent repellency, prevention of attachment and acaricidal efficacy against *D. reticulatus* within one day of administration to dogs and the effects continue for 4 weeks.

These results suggest that in endemic areas of canine babesiosis, monthly applications of the new combination could prevent tick attachment in dogs and thus significantly reduce the potential for transmission of *B. canis* as well as other tick-borne diseases.
